# Biotechnologies that empower transgender persons to self-actualize as individuals, partners, spouses, and parents are defining new ways to conceive a child: psychological considerations and ethical issues

**DOI:** 10.1186/s13010-018-0054-3

**Published:** 2018-01-17

**Authors:** Agnès Condat, Nicolas Mendes, Véronique Drouineaud, Nouria Gründler, Chrystelle Lagrange, Colette Chiland, Jean-Philippe Wolf, François Ansermet, David Cohen

**Affiliations:** 1 Service de Psychiatrie de l’Enfant et de l’Adolescent, Hôpital Pitié-Salpêtrière, Paris, France; 2 CESP INSERM 1018, ED3C, université Paris Descartes, Paris, France; 3 Équipe d’accueil CLIPSYD EA 4430, Connaissance, langage, modélisation (ED 139), université Paris Ouest–Nanterre La Défense, Nanterre, France; 4 CECOS Paris Cochin, Hôpital Cochin, Paris, France; 5 Lien POPI (périnatalité, orientation psychanalytique et institution), centre Dominique Mahieu-Caputo, Paris, France; 6 Service de Psychiatrie de l’Enfant et de l’Adolescent, Université de Genève, Hôpitaux Universitaires de Genève, Genève, Switzerland; 7 Institut des Systèmes Intelligents et de Robotiques, Université Pierre et Marie Curie, Paris, France

**Keywords:** Transgender persons, Gender dysphoria, Assisted reproduction technologies, Gender transition, Ethics

## Abstract

Today, thanks to biomedical technologies advances, some persons with fertility issues can conceive. Transgender persons benefit also from these advances and can not only actualize their self-identified sexual identities but also experience parenthood. Based on clinical multidisciplinary seminars that gathered child psychiatrists and psychoanalysts interested in the fields of assisted reproduction technology (ART) and gender dysphoria, philosophers interested in bioethics, biologists interested in ART, and endocrinologists interested in pubertal suppression, we explore how new biotechnical advances, whether in gender transition or procreation, could create new ways to conceive a child possible. After reviewing the various medical/surgical techniques for physical gender transition and the current ART options, we discuss how these new ways for persons to self-actualize and to experience parenthood can not only improve the condition of transgender persons (and the human condition as a whole through greater equity) but also introduce some elements of change in the habitual patterns of thinking especially in France. Finally, we discuss the ethical issues that accompany the arrival of these children and provide creative solutions to help society handle, accept, and support the advances made in this area.

**Electronic supplementary material:**

The online version of this article (10.1186/s13010-018-0054-3) contains supplementary material, which is available to authorized users.

## Background

Today, biomedical technologies enable persons with fertility issues to conceive. In the field of gender dysphoria (GD), they have allowed persons to change sex and adopt the gender they identify with [1]. Additionally, assisted reproduction technologies (ARTs) in the 80’s has impacted family law and created new ways for transgender persons to participate in parenting. Furthermore, the fight for equality and non discrimination has led to a better recognition of the rights of Lesbian-Gay-Bisexual-Trans-Intersex-Questioning (LGBTIQ) including in family law.

This paper is based on clinical multidisciplinary seminars that gathered child psychiatrists, psychoanalysts, philosophers, biologists, and endocrinologists interested in GD and ARTs during the year 2016, in which we shared our experiences and read numerus texts on this issue. We aimed at tackling the novel issues raised by those new ways for couples (in France ARTs are not available for single person), including transgender persons, to access fertility services in order to conceive a child (see Additional file 1: Glossary). Here, we first reviewed [1] the various medical/surgical techniques for gender transitioning and [2] the ART options that make new ways of conceiving possible. To this aim, we performed 2 theme searches on the last 10 years (2007–2017) in Pubmed and Psychinfo databases. The first on gender dysphoria used the following key-words (*gender dysphoria and transition, gender dysphoria and ethics, gender dysphoria, LGBTIQ health, transgender people and transition, transgender people and ethics, puberty suppression, gender reassignment*) and selected articles referring to self-actualization and ethical issues. The second on ARTs used the following key-words *(transgender people and assisted reproduction technology, transgender people and reproductive issues, gender reassignment and assisted reproduction, fertility preservation, uterus transplantation, artificial gametes, transgender people and fertility, assisted reproduction technology and ethics, reproductive wish and trans)* and we selected articles focusing on transgender persons and procreation and ethical issues. We also added key references on GD and ART that had been outlined by our experts during the seminar. These selected texts were discussed at the clinical multidisciplinary seminars.

Then, we pounder how these new opportunities, in addition to bringing a real improvement to the lives of transgender persons, would introduce some elements of change in patterns of “traditional thinking”. Finally, we discuss according the Beauchamp and Childress Principlism [2] the ethical issues that accompany the arrival of these “children of science” [3] and provide creative solutions to help society come to terms with the advances in this area.

### Gender dysphoria

It is now possible for persons who perceive themselves as transgender to align their anatomical attributes in order to be closer to the gender with which they identify. Social and cultural perceptions of transgender affirmation have evolved (Additional file 2 Supplement-e1). Today, the concept of sex is biological whereas the meaning of gender refers to two concepts [4]: ‘gender role’ which refers to what one says or does to disclose one’s status of boy/man or girl/woman, and ‘gender identity’ as the intimate sense of belonging to one’s sex [5, 6]. Gender dysphoria (GD) refers to a marked incongruence between one’s experienced/expressed gender and one’s assigned gender, leading to clinically significant distress [7]. The term “transsexualism” has been used in the past to describe persons who have social transition to another gender. This term has largely fallen out of favor and is considered pejorative by many.

If one’s identity is constructed by a “knot” among several dimensions (e.g., the real body, the body image, and the symbolic dimension), hormonal treatment and surgical transformation help the persons experiencing GD [8] reduce the discrepancy between these dimensions, so they can flourish as human beings. In doing so, they are accessing health as it is defined by the World Health Organization (WHO) that is: “*a state of complete physical, mental, and social well-being and not merely the absence of disease or infirmity*” [9].

The hormone-surgical transformation allows persons with GD to correct their anatomical structure to be closer to the gender with which they identify [1]. Typically, transgender men (Female-to-male, FtM) use hormonal transformation (most commonly progestins or GnRH agonists for a short period of time early in hormonal therapy, then testosterone through the end of their life) and surgical transformation (subcutaneous mastectomy, creation of a male chest, salpingo-oophorectomy and hysterectomy, phalloplasty or metaiodoplasty, vaginectomy, scrotoplasty, implantation of erectile and/or testicular prostheses). For transgender women (Male-to-female, MtF), hormonal transformation involves taking estrogen and anti-androgens and then undergoing surgical transformation (penectomy, orchiectomy, vaginoplasty, clitoroplasty, vulvoplasty, augmentation mammoplasty) [10–12]. Not all transgender persons choose to undergo hormonal or surgical interventions; some only choose a subset of these available interventions. Transition refers broadly to the development of one’s gender expression congruent with one’s internal gender identity, and can include any, some or all of the following –legal name changes, changes in appearance and behavior, medical therapies, surgical therapies – but does not require any of these changes.

For fifteen years, pubertal suppression (GnRH analogue administration at Tanner stage 2) has led to better results both physically and psychologically by avoiding internal strangeness and social isolation or rejection. It prevents the appearance of secondary sexual characteristics and enables the person to go through the real-life experience transition at school under better conditions [13]. The World Professional Association for Transgender Health (WPATH) and the US Endocrine Society, recommend this early management of GD at the stage of pubertal development Tanner 2 with pubertal suppression and, possibly, hormonal transformation with the administration of cross-sex hormones at the age of 16 [14, 15] while some medical centers currently advocate for the use of cross-sex hormones before the age of sixteen [16]. With this approach, the results are quite convincing in terms of physical appearance, urinary function, and even genital function, although there are still problems of urethral strictures and genital function is not yet fully satisfactory in the case of female to male genitalia surgery. To date, it remains technically impossible to ensure the capacity to procreate after one’s experienced-transition with one’s own genitalia. For years, transgender people had to choose between the transition to the desired gender and the ability to procreate since both hormonal and surgical therapies lead to the loss of reproductive potential. However, new advances in ARTs have allowed many persons to conceive children with or without their own gametes. Consequently, it is technically possible today to help transgender people not only to procreate but also to conceive children with their own gametes.

### Assisted reproductive technology

Although this issue is debated in the legal literature, according to the authors which papers we analyzed, both the United Nations Universal Declaration of Human Rights (1948) [17] and the WHO [9] recognize that every person has the right to procreate. Advances in ARTs have enabled persons with fertility issues to access to procreation [18]. Since the first in-vitro fertilization (IVF) [19], it became possible to disjoin fertilization and sexuality in terms of both time and place. ART has improved steadily; the freezing of embryos allows the birth of a baby from an embryo long after conception, microinjection enables fertilizing an egg with a sperm that was itself insufficiently fertile, and the general process allows future parents to choose among spermatozoa those that appear to be the best candidates for the fertilization process. It has also become possible to mature premature oocytes in vitro or to inject spermatids (immature spermatozoon) in oocyte. Gamete conservation techniques have also progressed; oocyte cryopreservation [20] has been improved by vitrification, and testicular pulp and ovarian tissue preservation are now performed (although the ability to use testicular tissue and ovarian tissue after their preservation remains experimental) [21–23]. These techniques have helped preserve the fertility of persons who had to undergo potentially sterilizing treatment (e.g., chemo), and of persons suffering from Klinefelter syndrome [24]. Although donor sperm inseminations (DSI) were already performed well before the possibility to freeze gametes, the creation of sperm banks in the 1970s, followed by the development of oocyte preservation techniques, led to the possibility of third-party procreation.

Finally, the use of a gestational carrier was made available by some clinics to women who were unable to carry a pregnancy and later for women who did not wish to carry a pregnancy, single men and gay couples, but it is also socially and legally regulated differently at the local or national level. These disparities are promoting the so-called reproductive tourism market.

Recently, for the first time, a baby was born from a transplanted uterus [25]. Future procedures seem focused on the production of synthetic gametes derived from somatic stem cells and embryonic stem cells. Such gametes have successfully been used to produce live offspring in mice, and research in this area is ongoing [26, 27].

The World Professional Association for Transgender Health (WPATH) Standards of Care recommend discussing fertility options with patients prior to any treatment or medical/surgical interventions [11]. The current options in fertility preservation include the cryopreservation of embryos, oocytes or ovarian tissue for transgender (FtM) men, the cryopreservation of sperm collected through ejaculation or direct testicular extraction, and the cryopreservation of immature testicular tissue for transgender (MtF) women [28]. Recent research shows that in a context where fertility preservation is proposed to transgender adolescents before transitioning, utilization rates of fertility preservation are low [29, 30]. By contrast, a small majority of adults transgender men and transgender women would actually have cryopreserved their gametes, or would have seriously considered doing it, if the technique had been available [28, 31].

### New ways to conceive a child

Biomedical advances enable Trans-persons to become parents outside the context of adoption. In this section, we detail all possible configurations of accessing to parenthood given new ARTs methods. This implies distinguishing the social father, the social mother, the genetic mother (oocyte donor), the genetic father (sperm donor), and the gestational mother. These configurations are not authorized in many countries, but their social implications are discussed at the end of this section.

**Fig. 1:**
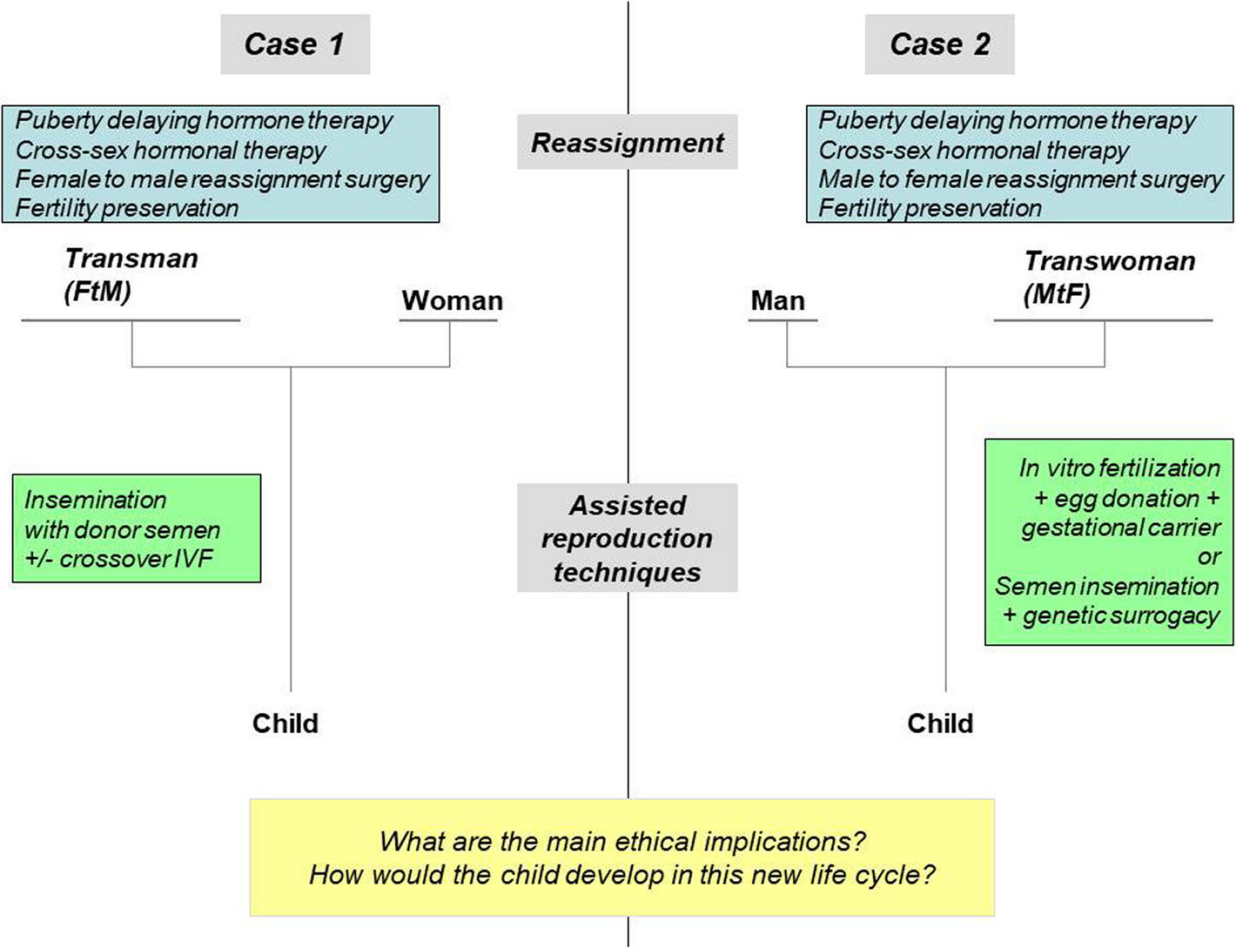
Ways of conceiving for trans-persons: configurations in heterosexual couples (see also Additional file 3: Figure S1 and Additional file 5: Figure S3 to account for fertility preservation)

Heterosexual couples in which the male partner is a transgender man may turn to ART by DSI (Fig. 1, case-1). In contrast, transgender women who have a male partner can procreate with the help of a gestational and genetic mother or the help of a gestational mother and the donation of oocyte from third party (Fig. 1, case-2). In case-1, if the transgender man cryopreserved his oocytes/ovarian tissue before transitioning, the couple can discuss cross-over IVF (the transgender man provides oocytes that are microinjected with sperm from a male donor to obtain embryos, which are transferred to his female partner). This technique is commonly performed in lesbian couples. In case-2, preserved semen from the transgender woman can also be used in IVF of the donated oocytes for all or part of the offspring (see Additional file 3: Figure S1 and Additional file 4: Figure S2).

**Fig. 2:**
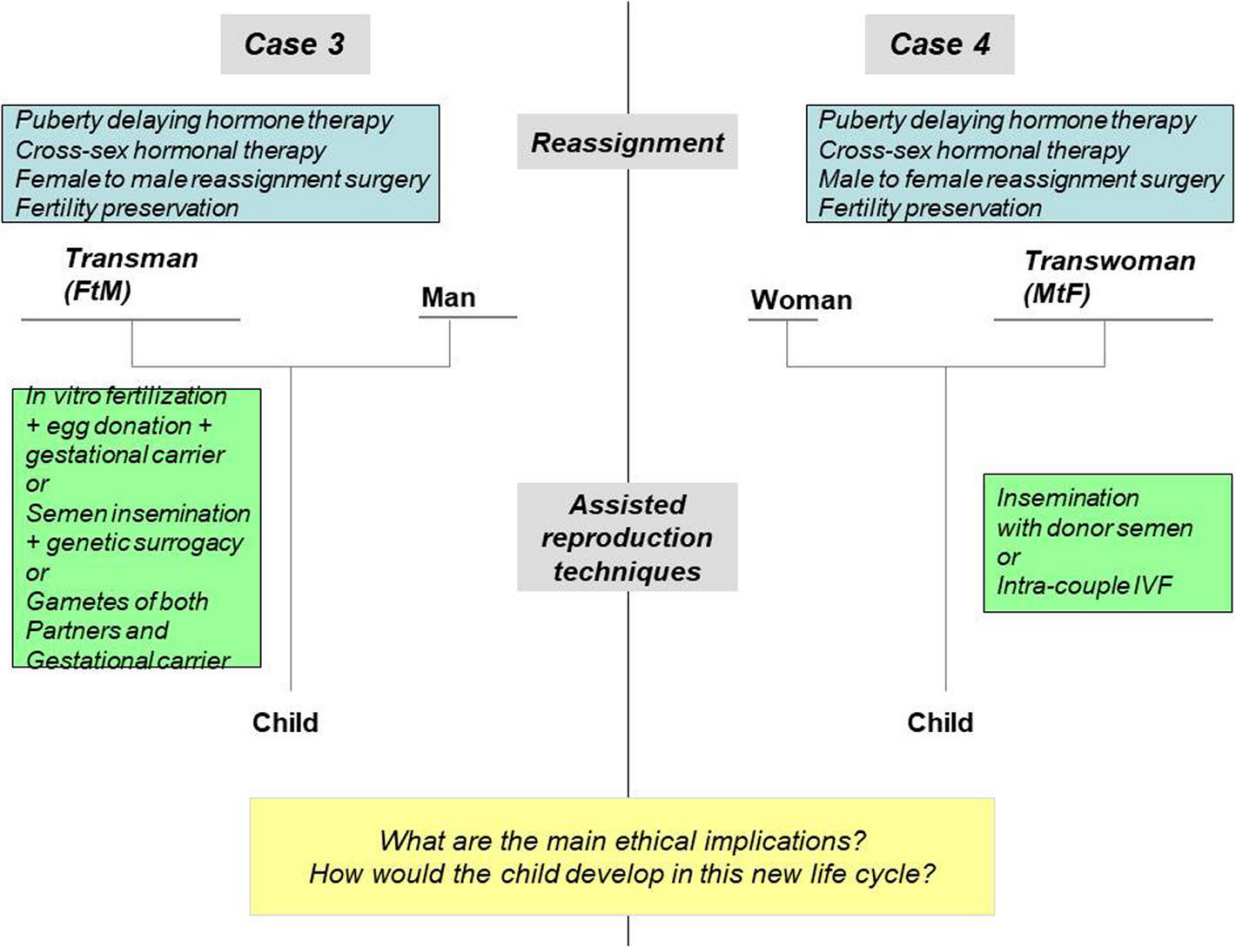
Ways of conceiving for trans-persons: configurations in homosexual couples (see also Additional file 4: Figure S2 and Additional file 6: Figure S4 to account for fertility preservation)

For gay couples in which one partner is a transgender man (Fig. 2, case-3), one option is to use the help of a surrogate mother. If the transgender man cryopreserved his oocytes/ovarian tissue before transitioning, IVF can be performed with the gametes of both parents and further assisted by a surrogate mother. In that configuration, one of the fathers will be the genetic father; the second will be the genetic mother; the surrogate mother acting only as a gestational carrier. If this transgender (FtM) man has not had his ovaries and uterus removed despite being recognized by law as a man, he can choose to gestate the child. As a consequence, he will not only be the father by law but also the genetic mother and gestational mother of the child, his partner could be the genetic father or in case of sperm donation, the second parent legally recognized. Lesbian couples in which one partner is a transgender (MtF) woman (Fig. 2, case-4) may turn to ART by DSI. If the transgender woman preserved semen before transitioning, this semen may be used in an intra-couple IVF, so the child would be the genetic child of both his parents. One of the child’s mothers is also the genetic mother, and the second is not only a mother legally but also the genetic father (see Additional file 5: Figure S3 and Additional file 6: Figure S4).

To date, not all of these options are currently accessible. In France, for example, surrogacy is prohibited by law, and ARTs is only available to heterosexual couples [32]; the only option available for transgender people is DSI for heterosexual couples with a transgender (FtM) man (Fig. 1, case-1). Fertility preservation is not provided to transgender people by French fertility services. Even in countries without legal/administrative barriers, trans-people report negative experiences with ART-service providers, including problems with clinical documentation; negative impact of normative providers; and heteronormative assumptions and the refusal of services for prospective transgender clients [33]. Although societal changes and advances in medicine permit such new ways to conceive a child, they also introduce new changes in the ancestral logic of conceiving. The traditional bounds (also called “sexuation” by some authors [34, 35]) among gender identity, sexuality, conception, gestation, procreation, and filiation are deeply challenged [36]. If the spread of contraception over the last fifty years has caused an effective separation between sexuality and procreation, the current disruptions in conservative thinking are going much further.

In the past, gender was mainly considered a discrete variable with two dimensions (three in a few cultures) [37–40]. It was one of the major markers in the construction of identity and relationship. Currently in our experience, many teenagers consider gender to be a continuous variable, along a spectrum from male to female, including non-gendered. Similarly, one’s sexual orientation or choice of sexual partner is no longer binary, heterosexual or homosexual, or even bisexual or asexual but can be located somewhere else on this gender continuum (bringing to mind the scales of bisexuality [41]). It even comprises the entire continuum, such as those adolescents naming themselves ‘pansexual’ or ‘genderblind’ [42]. In relation to parenthood, many adolescents from Western societies have grown up in single-parent families or step-families, so the idea of being a single parent or, on the contrary, forming a family composed of three or four individuals as parents does not imply negative social implications for them. With ARTs, it is now possible to conceive a child as a single parent. In other configurations, five individuals can be involved in conceiving a child (e.g., homosexual couples including a trans-man (case-3, Fig. 2) with embryo donation and the conception involving another man and another woman).

With surrogates, the woman giving birth to a child is not always considered by the law as the mother even when her oocytes were used. However, a man is always considered the biological father (which is different from being considered as the legal father) if ARTs used his own semen. In case-2, the mother is neither the woman who bore the child nor the one who provided her oocytes, hence her designation as “social mother”. Although semen comes from fertility preservation, as shown in case-1, the spermatozoa come from a person who is socially a woman, and some would say the genetic father is a woman (Fig. 1). Furthermore, with embryo preservation, a child may be born decades after its conception, with its parents belonging to a different generation than the man and woman who donated their gametes or their embryo (Additional file 3: Figure S1, Additional file 4: Figure S2, Additional file 5: Figure S3, Additional file 6: Figure S4).

### Some insights into what drives fear and anxiety

**Fig. 3:**
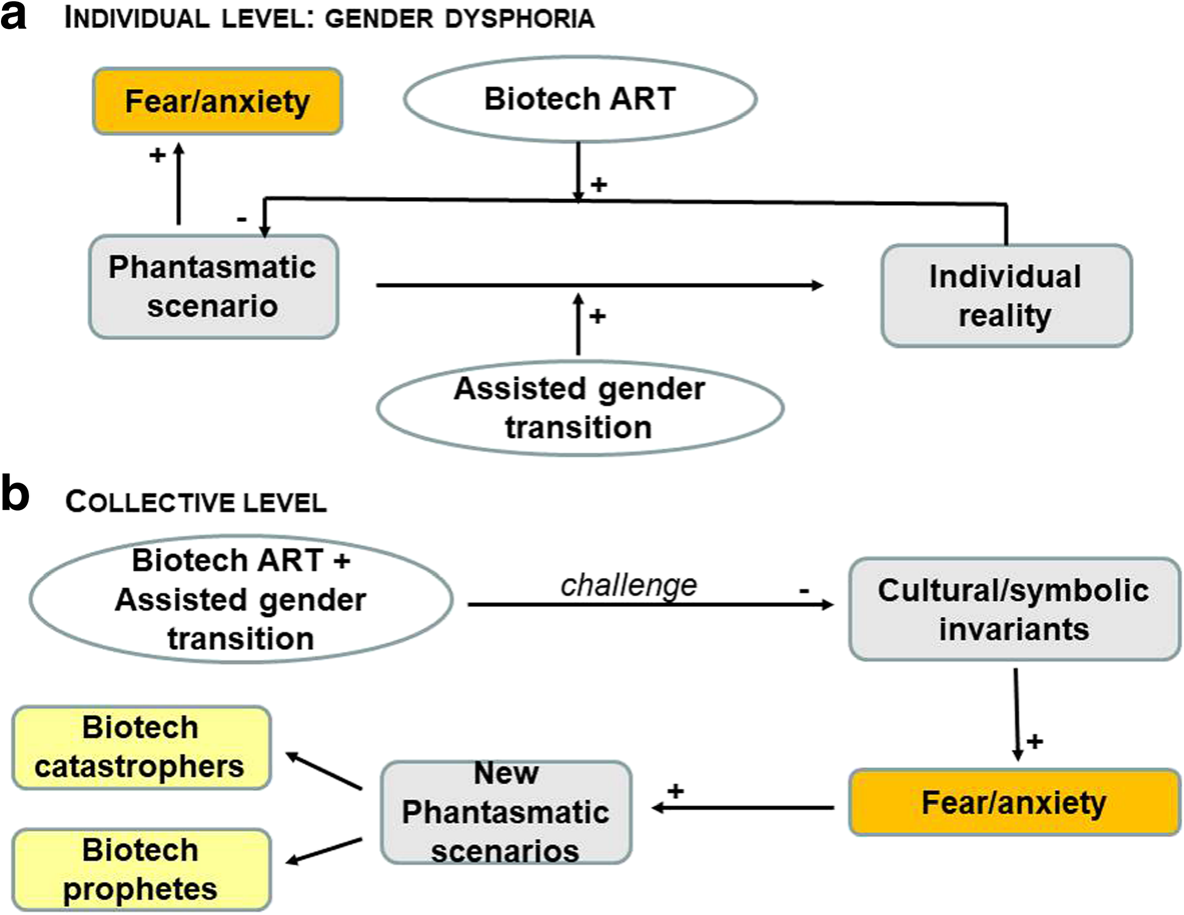
Schematic representation of fear and anxiety in individuals with GD (**a**) and at the level of cultural/symbolic invariants of monotheistic societies (**b**)

At the individual level for persons with GD, fear and anxiety come from the discrepancy between one’s own reality (e.g., the biological/birth gender) and one’s own imagined alternate scenario (e.g., transitioning to the wanted/opposite gender) [43]. Gender transition helps persons with GD resolve this discrepancy, and ART may assist them in achieving new imagined and desired scenarios, such as having a child. As a consequence, when transition is achieved, persons with GD will feel relieved (Fig. 3a).

**Fig. 4:**
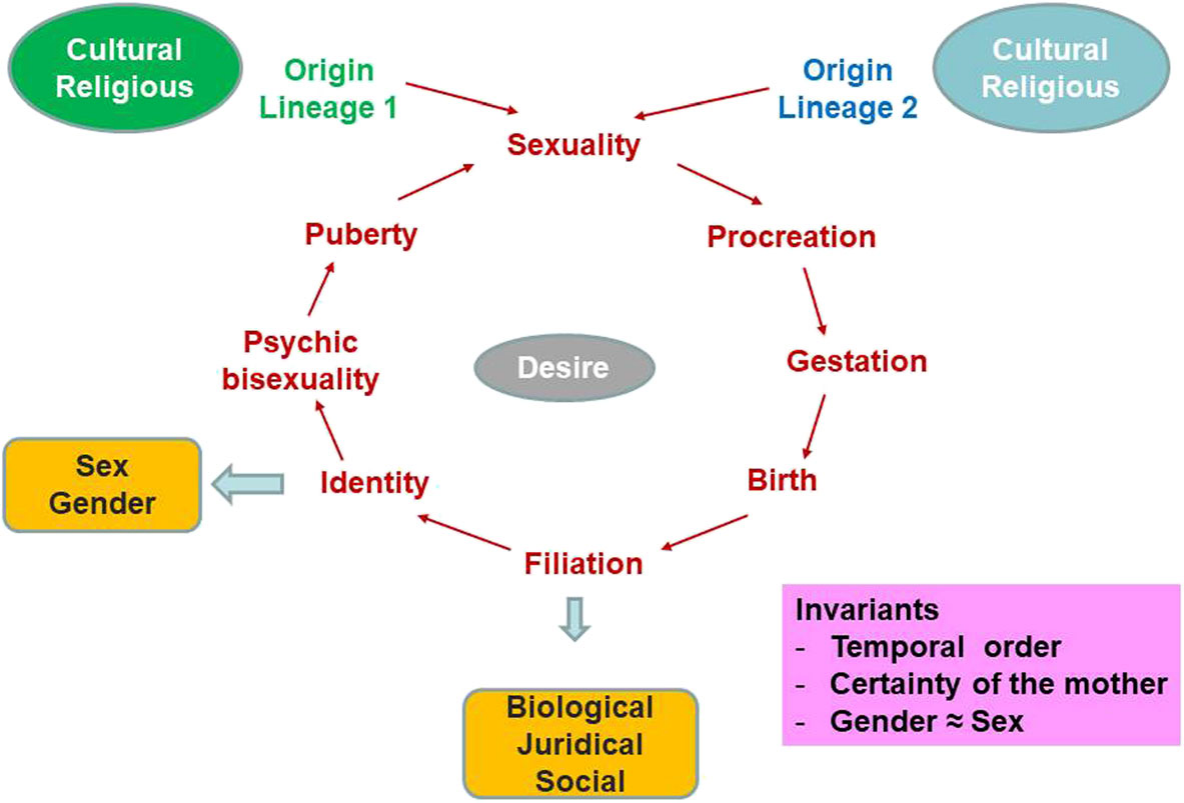
The “traditional” life course in monotheistic societies

At a collective level, the above-mentioned new configurations of child conception challenge some variants of sexuality or relations that are anathema to some cultural or religious communities, which we believe may explain some of the individual and collective struggles in accepting these new forms to access to parenthood. Origin is a complex and demanding issue for anyone. Basically, it refers to the question: “Where was I before being in my mother’s womb?” The ancestral way to conceive a child refers to two equal lineages (that of the mother and that of the father) and the traditional transition steps from conception to adult sexuality (Fig. 4). In each culture, both the imaginary and symbolic orders enabled generations to manage this fundamental gap. Figure 3b is an attempt to represent how fear and anxiety are driven at a collective level. The disturbances created by new ways to conceive a child may create anxiety or shock individual and collective thinking. The assisted medical transitions of people with GD and assisted reproductive technology (ART) challenge several cultural/symbolic invariants, including [1] the temporal order, [2] the certainty of one’s mother, and [3] the parallel link between sex and gender. Anything that destabilizes these “traditional” invariants may create discomfort and apprehension. Although it is not the scope of our paper, we want to highlight some important dimensions that could be challenged at an individual level because of the discrepancies between individual beliefs and the (changing) symbolic repertoire of a society: one-self-identity, desire, procreation, filiation, and the relationships between being a man and a father or a woman and a mother.

This fear of the unknown and the lapsing of traditional markers promote the emergence of new fantasies in two opposite directions: bio-catastrophists vs. techno-prophets [44]. The former think that science serves as driving force bringing about apocalyptic times through its destruction of norms and traditional modes and understandings of the meaning of life, with severe consequences for society and, ultimately, the resulting end of the human species. The latter believe that science offers the promise of a paradisiac future, a new redemptive era with a pure incorporeal spirit emerging from thinking machines.

Many experts in France remain critical of the idea of procreation after gender transition, and about preservation of fertility to parent genetically related children [32]. The current legislation in France allows limited opportunities for transgender people to do so. Fertility preservation is actually quite rare among adolescent patients in the US [29, 30]. French teenagers and young adults who visit our gender clinic, interact on forums with peers from other countries. This process leads some of them to ask for the preservation of their fertility before gender transition. Although the use of their gametes is currently impossible, they know that cryopreservation techniques could enable the use of their gametes in the future, if the law changes. As practitioners, these new demands imply ethical investigation, not just opinions on what is right or wrong.

### Biomedical ethics through the lense of Beauchamp and Childress’ principlism

Biotechnologies occasionally go beyond the limits of our thinking or imaginative capacities, and bring ethical implications of medical and societal aspects on the one hand and of individual and collective interests on the other. These new ways to conceive a child crystallize several questions regarding [1] puberty suppression and early school transition, which are largely allowed in several countries (e.g., USA, Canada, UK, The Netherlands, Belgium, Germany) but are not yet in others (e.g., France, Italy, Spain); [2] ARTs, including technologies directed at transgender people; and [3] the preservation of fertility. This last theme is relevant to other medical situations (e.g., chemotherapy) but raises specific issues with transgender people. On each level, the questions will be studied according to the approach of medical ethics developed by Childress and Beauchamp ie Principlism [2, 45].

#### Beneficence and non-maleficence

Two major objections have been raised regarding these principles. The first is the purported “unnatural” characteristic of these practices and the inability to reconcile them with medical ethics [46]. However, for most authors, this ‘argument from Nature’ is deeply problematic because there is no common agreement that would enable us to clearly distinguish between natural and unnatural conditions/actions. It is “natural” for humans to manipulate “nature” in order to accomplish our desired ends. Therefore, an objection based on something’s being “unnatural” is deeply problematic [47–49]. The second is a consequentialist concern: Are there any harmful effects on the individuals, on unborn children, on third-party stakeholders, or on society?

Regarding pubertal suppression and early social transition in countries that do not allow it, clinicians think that providing such support will encourage the youth to undergo a potentially burdensome transsexual evolution because this course of life is particularly difficult due to the life-long hormonal treatment, the heavy surgical interventions and the social prejudice. However, the international literature gives evidence that gender identity rarely changes after the beginning of puberty and that providing such support significantly improves the quality of life and diminishes self-discrepancy feeling, depression, suicide attempts, social discomfort, truancy due to bullying, and social exclusion [13, 50]. Moreover, pubertal suppression followed by hormonal transformation improves the quality of life in adult age, thereby allowing optimal physical results by preventing the appearance of secondary sexual characters of the birth sex and avoiding other interventions (e.g., mastectomy for trans-men) [51]. However, the use of hormones could also modify the trajectory of adolescent libido and desire. To our knowledge, this possible consequence has not yet been discussed.

With procreation being possible via ART, its benefit is the possibility of experiencing a previously unavailable form of parenthood. Transgender people have a desire to become parents [52, 53]. It is a self-flourishing project, transforming oneself and one’s identity. Accessing parenthood contributes to realizing one’s desired sense of self and gaining recognition in society [54]. For transgender people, it may be even more important; becoming a mother can contribute to expressing and consolidating a female identity, and becoming a father can contribute to expressing and consolidating a male identity [49].

Some may question the welfare of the offspring because such trans-family forms are often confusing to many. The sparse research on the psychological well-being of transgender people’s children suggests that there is no support for the idea of any adverse impact on children. In four studies, in which children were born before their parent’s transition [55–58], none of them developed gender identity problems. In Freedman’s study, only one 18-year-old child suffered from depression, and most of the children participants did not seem to have major psychosocial problems. Nevertheless, some children experienced difficulties in their relationship with peers (33%) [57]. In White’s study, 19 of the 55 children suffered from psychiatric disorder, including depression (*n* = 7) and Attention-Deficit/Hyperactivity Disorder (*n* = 4). The disorder started prior to the parent’s transition in 12 children and after the transition in 7 children. There were fewer difficulties in the absence of parental conflict and when the child was younger at the time of the transition [58]. Green published findings on three cross-generational GD families [59]. However, there is no known causative link in the cross-generational cases, and the cross-generational families are few in number compared to the total population of transgender people participating in parenting [57]. Our clinical experience of children with trans-parents confirms the determinant impact of both parents’ ability or inability to put into words the experience of transition on their children welfare. Contrary to young children, we notice that teens also report social difficulties in connecting with others, difficulties that were accrued with their peers and family criticisms of their trans-parent.

Children who are conceived by transgender people after their transition do not have to adapt to a new parental identity and are less confronted to socially aversive reactions. To our knowledge, only one study has been issued so far on this topic: 52 children born between 2000 and 2015, from DSI for couples with a transgender man, have been followed every two years. The qualitative results show that children have a normal development without any major psychological morbidity or GD [60]. Most of the children participants knew that they were born by third-party ART and that their fathers were born as females. The follow-up study is ongoing with a quantitative standardized evaluation. In Ghent, another ongoing study is investigating children of MtF and FtM parents using the ‘high risk of serious harm’ standard [47].

Another inquiry is the potential risk for transsexual parents in waiting, given psychiatric morbidity [61]. Research showed that if trans-persons are treated well after their transition, their welfare is improved and their psychiatric morbidity does not differ from the general population [13, 62, 63]. Because GD is no longer labeled a mental disorder, when considering any factor relevant to an ethical decision, the well-being of the applicants themselves should be assessed by using the same criteria as for heterosexual couples. It seems that the risks for transgender parents are mainly social because transsexual parenthood may encounter severe criticism and opposition. The medical risks for fertility preservation are very low; trans-men may undergo ovarian stimulation or provide the ovarian tissue that will be extracted during the sex transition surgery. No additional surgery is necessary. The medical risks induced by ARTs are those caused by the techniques, such as the risk of ovarian hyperstimulation syndrome for the female partner when ovarian stimulation is required. However, if uterus transplantation becomes available in the future, the specific risks for trans-women should be thoroughly evaluated.

The risks for potential oocyte donors (inherent to ovarian stimulation) and those for gestational mothers (inherent to pregnancy, delivery, and eventually their own families) have led some countries to prohibit surrogacy. Human body exploitation is considered a form of modern slavery [64]. According to the limited empirical data, most oocytes donors and surrogates consider these risks to be manageable if one adheres to the guidelines developed to minimize these risks [65]. In addition to these risks, it is important to consider how the involvement of other adults from the so-called social mother and father will impact the dynamic and the family system for the incoming child. For example, should we promote anonymous donation? Or on the contrary, should the child born have the right to know his/her genetic father or mother if he/she decides to? Should we discuss new forms of families and authority relationships with multiple fathers or mothers?

Finally, at the collective/societal level, is there a potential harm to society as a whole? This issue follows the larger debate regarding how human activities participate in the evolution of the species. In the case of transgender people parenthood, the societal harm often remains unspecified (e.g., religious/moral critique around the undermining of the nuclear family considered to be harmful – a deontological objection disguised as a consequentialist objection) [47, 66]. We wonder whether the new fantasies that are being articulated are related to fear of the unknown, as noted above. However, society is constantly changing, and these new ways to conceive a child are also the product of social developments. Transgender people represent a very small population, and as such, their access to procreation should not cause major social upheaval. We thus wonder whether there may be benefits to society in welcoming this diversity as enriching and thus require more thought about these issues. Some of the most interesting questions for bioethics are inquiries opened by LGBTIQ people in matters of identities, familial relationships, and stance before the government [67]. Allowing the construction of an identity that is not based on a rejection or a camouflage of the norm should be a major concern for democracies, as should the struggle against social inequality between genders. Further, sociologists wonder if those societal changes may contribute to racialized, stratified possibilities of child bearing and parenthood; in France, only one class of transgender people (case-1) is allowed to access ART; in the US, access is restricted by medical insurance infertility categories regarding infertility and to those who can afford it [54].

#### Autonomy

In the decision to engage in an early social transition at school, puberty suppression, or hormonal transformation, the autonomy of the child’s or adolescent’s must be raised. His/ her capacity to give a consent has to be determined. Furthermore, what do we decide when the parents and the child or adolescent disagree? The dilemma is rather similar to that found in other medical care cases where consent from the parents is not obtained, despite the child or adolescent’s assent.

Reproduction is an important element of the autonomy of people. If the right to procreation has been traditionally granted to heterosexual couples, the meeting of ART with LGBTIQ has changed the way of thinking about reproductive rights for non-heterosexual couples. It is evolving towards recognizing LGBTIQ the same right to procreation. In France, homosexual individuals have access to adoption but not to ARTs. Trans-men have access to ARTs because they are considered as men equal to every other man. Trans-women, however, do not have access to ARTs due to the legal prohibition of surrogacy and ARTs in homosexual couples. As a consequence, there is no legal way for trans-women to procreate. However, trans-parenthood did not wait for progress in biotechnologies or for non discriminatory arguments to be made in order to access equally to procreation, and autonomy has been reached by transgender people using their own means. Using “do-it-yourself” processes, transgender people have long become parents, despite the limitations in accessing ART [68, 69]. Since the literature review shows that the right to procreate is accepted as a universal human right, the respect of a person’s autonomy leads to granting each individual the right to reproduce. This right is relevant even in non-standard family forms and regardless of gender identity status [47, 70]. In the same way, transgender people should be counselled on reproductive issues by professionals, prior to initiating treatment, to have a clear overview of the treatment effects, the preservation possibilities, and what to expect [29–31].

#### Justice

This principle stipulates that related cases should be treated in a similar way, except when there is a morally relevant difference between them. What could be a morally relevant difference between trans-parenthood and heterosexual parenthood? As noted above, the few data that have been published do not suggest negative consequences for either the child or the parents. Fertility preservation will be the only way to give birth to genetically related children after transition surgery for transgender people. Some argue that trans-men and trans-women are choosing a sterilizing treatment that is not necessary for their health. Therefore, they should not go through transition surgery if they wish to have children [32]. However, after listening to what transgender people say, hormonal and surgical transitioning does not seem to be a choice but on the contrary, an absolute necessity to achieve their identity construction process and attain the state of complete physical, mental and social well-being that defines health as defined by the WHO. Consequently, trans-persons should be considered in the same way as other patients, and there should not be an argument to demand a parenting project when fertility preservation is chosen by trans-adolescents, if such a parenting project is not required from adolescents suffering from cancer. At that age, most adolescents do not have such preoccupations and are not able to project themselves into the future.

Additionally, there should not be a discriminatory double standard with stricter evaluation criteria for the risk of harming the offspring. The issue of gender transition must not be allowed to mask any other factors relevant to ethical decision making; the couples should be assessed using the same criteria as heterosexual couples (e.g., why, in France, can trans-men become parents but trans-women cannot?). However, other relevant considerations, such as the interests of eventual surrogates to avoid human exploitation, should also be taken into account [47–71]. Finally, when there is no legal prohibition to ARTs for trans-men and trans-women, inequity may still arise in some countries due to the lack of support of ARTs by the medical insurance system [72].

## Conclusion

Medical advances allow transgender persons to go through a ‘sex change’ in the direction of their experienced gender. At the same time, ARTs and gamete preservation have forged new ways for persons to access parenthood, resulting in the introduction of elements of change in the conservative patterns of thinking about our origins. How could we address these new ways to conceive a child? We believe there are two options: [1] refusing it all because we cannot bear to think about it or [2] based on the idiosyncratic courses of action mentioned above, being creative and inventive witnesses of broader societal developments that can lead to societal progress in terms of human rights. Through the usage of “do-it-yourself” processes, transgender people are becoming parents; the second option is likely to be ongoing. We think that a better ethical debate could occur by dismissing opinions and adopting medical ethics standards derived from principlism to delineate risks and benefits at both the individual (parents in waiting and surrogate) and collective levels, on the one hand, and the anticipated empirical research, on the other. Despite societal opposition [73], more research is needed on the health of surrogate mothers, the long-term outcome of fertility preservation and hormonal suppression, and the developmental well-being of children from trans-parents.

## Additional files

Additional file 1: Glossary. (DOCX 15 kb)

Additional file 2: Supplement e1 [74]. (DOCX 15 kb)

Additional file 3: Figure S1. Heterosexual couple with a transgender man. Image 1. (JPEG 50 kb)

Additional file 4: Figure S2. Homosexual couple with a transgender man. Image 2. (JPEG 46 kb)

Additional file 5: Figure S3. Heterosexual couple with a transgender woman. Image 3. (JPEG 53 kb)

Additional file 6: Figure S4. Homosexual couple with a transgender woman. Image 4. (JPEG 53 kb)

## Abbreviations

ART: Assisted Reproduction Technology
ARTs: Assisted Reproduction Technologies
DSI: Donor Semen Insemination
FtM: Female to Male
GD: Gender Dysphoria
IVF: In Vitro Fertilization
LGBTIQ: Lesbian-Gay-Bisexual-Trans-Intersex-Questioning
MtF: Male to Female
WHO: World Health Organization
WPATH: The World Professional Association for Transgender Health
