# Supplementary material for: Biotechnologies that empower transgender persons to self-actualize as individuals, partners, spouses, and parents are defining new ways to conceive a child: psychological considerations and ethical issues
Source: Philos Ethics Humanit Med. 2018 Jan 17;13:1. doi: 10.1186/s13010-018-0054-3 (PMC5772725; doi:10.1186/s13010-018-0054-3)
Supplement: Supplementary file 1 — Glossary. (DOCX 15 kb) [file 13010_2018_54_MOESM1_ESM.docx]

**GLOSSARY**

**Egg/ oocyte donation:** Form of fertility treatment where a healthy woman donates eggs/ oocytes that can be fertilized by sperm from the recipient woman’s partner and the resulting embryos are placed into the recipient woman’s uterus.

**Fertility preservation:** Process of saving or protecting oocytes, sperm, or reproductive tissue so that a person can use them to have biological children in the future.

**Gender dysphoria:** discomfort or distress connected with one’s own gender incongruence (social, physical or both).

**Genetic mother/father:** The man or woman whose gametes led to the conception of the child, and thus which transmits its genetic heritage to the child.

**Gestational surrogacy /genetic surrogacy:** a woman chooses to carry a pregnancy for those who cannot carry a pregnancy to term without help. In gestational surrogacy, the pregnancy results from the transfer of an [embryo](https://en.wikipedia.org/wiki/Embryo) created by [in vitro fertilization](https://en.wikipedia.org/wiki/In_vitro_fertilisation) (IVF) using eggs that do not belong to the surrogate. Gestational surrogates are also referred to as gestational carriers. In genetic surrogacy, the surrogate herself provides the eggs and is therefore genetically related to the child.

**Hormonal suppression:** treatment by GnRH analogs that delays puberty and allows the suppression of further development of the secondary sexual characteristics of the natal gender.

**In vitro fertilisation (IVF):** Oocytes and sperm are taken from a couple and are incubated together in a dish in a laboratory to produce an embryo that is placed into the woman's uterus, where it may implant and result in a successful pregnancy.

**Insemination:** the placement of a man's sperm into a woman's uterus using a long, narrow tube.

**Transgender person:** transgender persons experience a gender identity that is different from the sex assigned to them at birth.
