# Supplementary material for: Biotechnologies that empower transgender persons to self-actualize as individuals, partners, spouses, and parents are defining new ways to conceive a child: psychological considerations and ethical issues
Source: Philos Ethics Humanit Med. 2018 Jan 17;13:1. doi: 10.1186/s13010-018-0054-3 (PMC5772725; doi:10.1186/s13010-018-0054-3)
Supplement: Supplementary file 2 — Supplement e1 [74]. (DOCX 15 kb) [file 13010_2018_54_MOESM2_ESM.docx]

**Supplement e1.**

Changes in wording from the 50ties:

1. Although the concept of sex is biological, the concept of gender, whose original meaning is grammatical, took on a new meaning with John Money in 1955 (3). He defines two concepts; firstly, ‘gender role’ as all one says or does to disclose one’s status of boy/man or girl/woman, and secondly ‘gender identity’ (first proposed by Evelyn Hooker as specified by Money (4) as the intimate sense of belonging to one’s sex (5).

2. The gender dysphoria syndrome was used in intersex adults about thirty years ago by Norman N. Fisk in order to account for the complexity of situations encountered at the transgender clinic, but also to place the focus on the expressed suffering of these people and justify the importance of providing care to them (7). The term ‘transsexualism’ was theorized by Harry Benjamin in 1953 (74), although the term had been used before by Hirschfeld (72).

3. Today, the term gender dysphoria (GD), as defined in DSM-5 (6) refers to marked incongruence between one’s experienced/expressed gender and one’s assigned gender, leading to clinically significant distress and/or social, occupational and other functioning impairment.

4. Transsexualism is an old term sometimes still used to define those who have a social transition to another gender. That is to say, they are recognized as belonging to the opposite (or another) gender to their birth-assigned gender. These people frequently receive hormonal treatment and surgery for physical gender transition, so that their bodies fit best with their feelings of belonging to the gender with which they identify (41). Today, the term transgender is preferred.
