# Supplementary figures and images for: Biotechnologies that empower transgender persons to self-actualize as individuals, partners, spouses, and parents are defining new ways to conceive a child: psychological considerations and ethical issues
Source: Philos Ethics Humanit Med. 2018 Jan 17;13:1. doi: 10.1186/s13010-018-0054-3 (PMC5772725; doi:10.1186/s13010-018-0054-3)

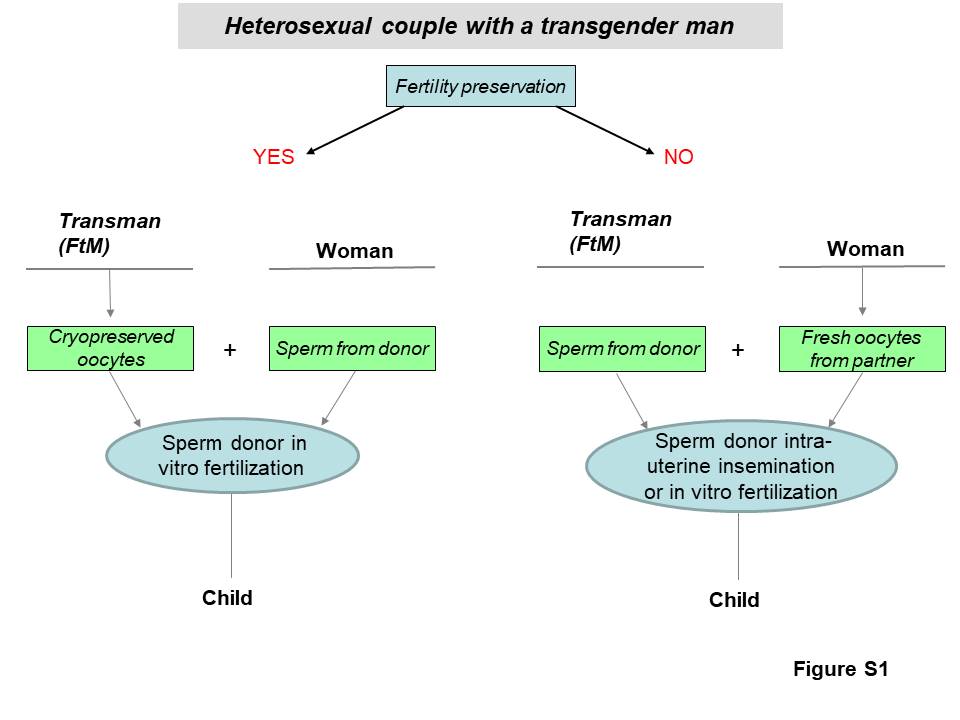

Supplement: Supplementary file 3 — Heterosexual couple with a transgender man. Image 1. (JPEG 50 kb) [file 13010_2018_54_MOESM3_ESM.jpg]

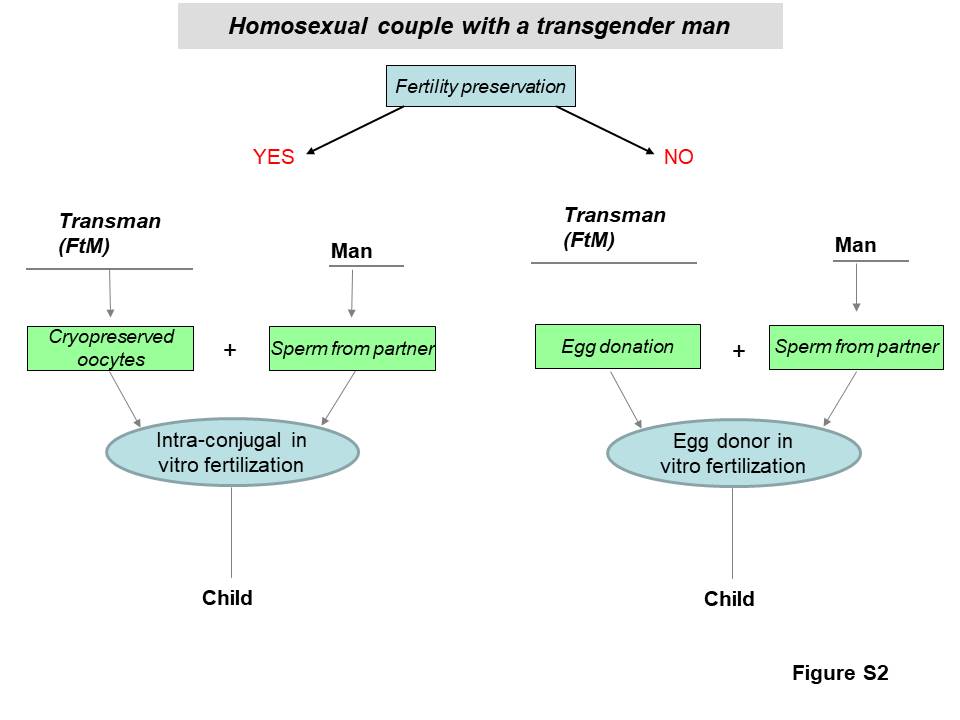

Supplement: Supplementary file 4 — Homosexual couple with a transgender man. Image 2. (JPEG 46 kb) [file 13010_2018_54_MOESM4_ESM.jpg]

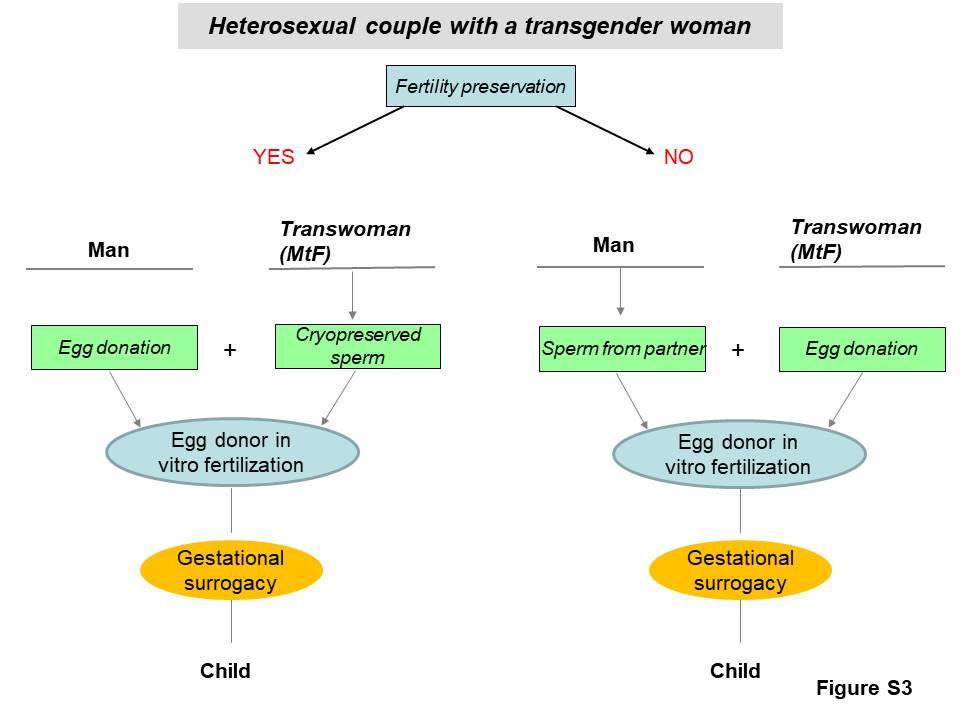

Supplement: Supplementary file 5 — Heterosexual couple with a transgender woman. Image 3. (JPEG 53 kb) [file 13010_2018_54_MOESM5_ESM.jpg]

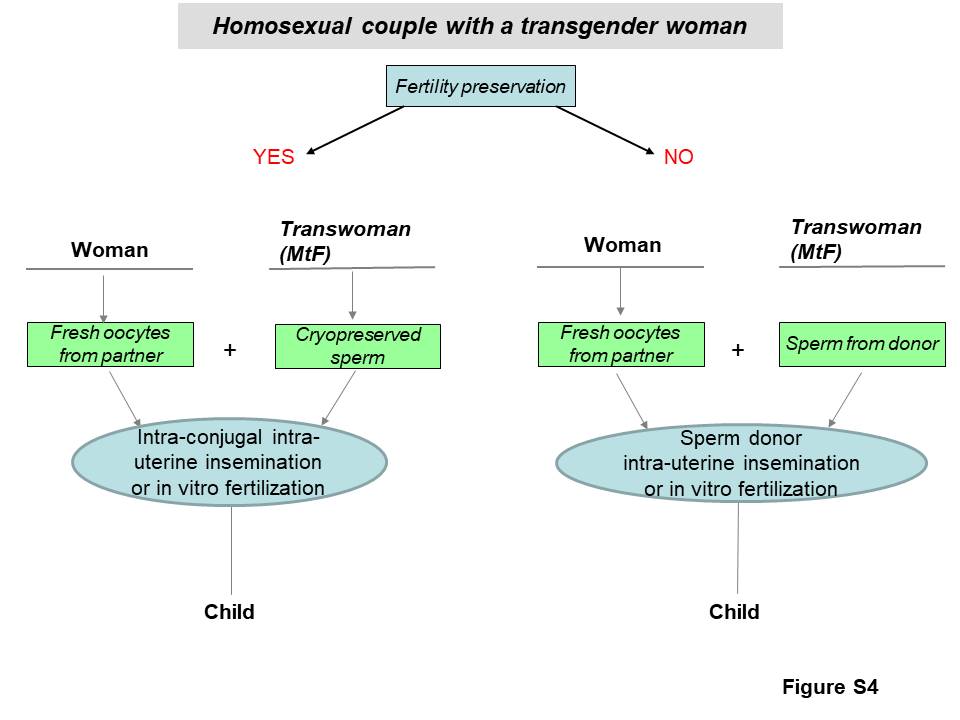

Supplement: Supplementary file 6 — Homosexual couple with a transgender woman. Image 4. (JPEG 53 kb) [file 13010_2018_54_MOESM6_ESM.jpg]
